# Analysis and Prospect of the Application of Wireless Sensor Networks in Ubiquitous Power Internet of Things

**DOI:** 10.1155/2022/9004942

**Published:** 2022-06-15

**Authors:** Li Cao, Zhengzong Wang, Yinggao Yue

**Affiliations:** ^1^School of Intelligent Manufacturing and Electronic Engineering, Wenzhou University of Technology, Wenzhou 325035, China; ^2^Intelligent Information Systems Institute, Wenzhou University, Wenzhou 325035, China; ^3^Key Laboratory of Intelligent Image Processing and Analysis, Wenzhou, China

## Abstract

With the rapid development of the economy and society, the low efficiency and high loss of the traditional power grid can no longer meet the growing social demand, and the power grid market is facing a reform. Smart grid, as a next-generation power system, it can effectively improve the performance of traditional power grids. The ubiquitous power Internet of Things (UPIOT) replaces the traditional grids with efficient, safe, reliable, and flexible new grids, improves the utilization efficiency of the grid, reduces the loss of the power grid in the transmission process, and can meet the needs of different types of markets and users. As an advanced information acquisition and processing technology, wireless sensor networks have been widely used in medical, industrial, agricultural, commercial, and public management fields. It is an important means to promote future economic development and build a harmonious society. In the power system, wireless sensor network technology can be widely used in many fields such as line fault location, real-time monitoring, smart meter reading, and relay protection. In this paper, the basic concepts and overall architecture of ubiquitous power Internet of Things are summarized. Then, we summarize the research status of the wireless sensor network in smart grid, including power equipment, line monitoring, smart grid wireless automatic meter reading, distribution network relay protection, power assets life-cycle management, power grid fault location, and power grid fault diagnosis. In view of the technical characteristics of wireless sensor networks, combined with the production links of power systems, the application framework of wireless sensor network technology in the power systems is constructed. The application of wireless sensor networks is prospected from the aspects of network development of relay protection, application research of smart substation, application research of power grid catastrophe, security protection of power system, and deep-seated ubiquitous power Internet of Things.

## 1. Introduction

The traditional power system monitoring and diagnosis system is realized by wired communication, but the wired monitoring system has the high installation and maintenance costs and inconvenient installation [1, 2]. Therefore, it is a good choice to adopt a cheap and easy-to-install wireless monitoring and diagnosis system [3]. Wireless sensor network technology has the characteristics of low cost, self-organizing network, and application-related and is very suitable for wireless monitoring and diagnosis systems of power grids [4–8]. As an advanced information acquisition and processing technology, the wireless sensor network has been widely used in medical, industrial, agricultural, commercial, public management, and other fields, and it is an important means to promote future economic development and build a harmonious society [9–11]. As the most flexible and extensible network structure in the wireless communication technologies, WSNs can support potential surveillance and control applications throughout the power network [12–14]. For example, on the generation side of the power system, the applications for WSNs include the monitoring thermal power plants, wind farms, photovoltaic panels, and various types of distributed generation equipment [15, 16]. In the power transmission, substation, and distribution side of power systems, the applications of WSNs include the monitoring overhead transmission lines, the underground transmission lines, wire towers, utility poles, and various substations and substation equipment. On the power side of the power system, the applications for WSNs include the ARM, AMI, and building energy management [7, 17].

The smart grid combines advanced technologies including sensing, measurement, control, and decision-making. On the basis of the integrated and high-speed communication network, the intellectualization of power grid is brought into full play [9]. Through the above means, the smart grid can achieve the goals of reliability, safety, and efficiency of the power grid, and at the same time, it can monitor and control each user and the nodes. The main objectives of the smart grid include the following: (1) encourage users to participate in the operation of power grid actively; (2) make effective use of all electric energy; (3) develop new products, services, and markets; (4) provide high-quality electric energy to meet economic needs; (5) provide the utilization rate of power assets to ensure the efficient operation of power network; (6) effectively resist various kinds of interference through automatic prevention, control, and repair; (7) have certain self-repairing ability; self-healing and robustness [18]. In smart grid, the real-time and reliable acquisition of grid operation status information is the key factor to ensure the reliable and efficient transmission of power from generation side to user side. Real-time monitoring, diagnosis, and protection of power system can prevent, mitigate, or avoid the impact of power equipment faults and natural disasters on the system. Therefore, using online real-time sensing technology to monitor the operation of power grid is the key to ensure the safe, reliable, and efficient operation of smart grid [19].

**Figure 1:**
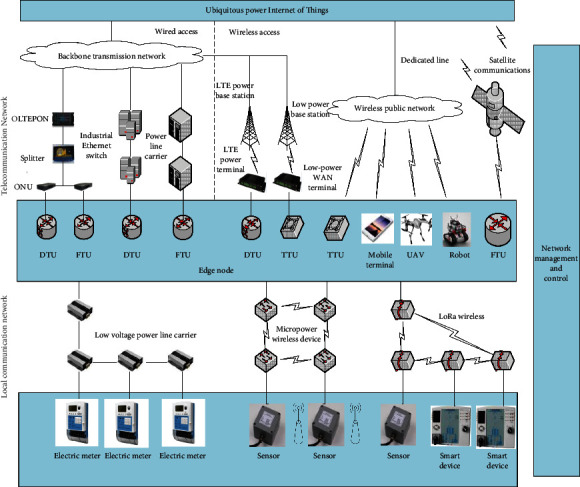
The schematic diagram of the ubiquitous power Internet of Things.

With the increasing proportion of new energy grid access and the deepening of power reform, the traditional power grid and power enterprises are facing greater external competitive pressure, while the traditional equipment is difficult to match the volatility of new energy, and the lack of interaction with the external environment makes it difficult for enterprises to meet more new demands of users [10]. The integration of smart grid and ubiquitous power Internet of Things is an indispensable link in building an energy Internet. It is extremely urgent to focus on building a ubiquitous power Internet of Things [20]. Ubiquitous power Internet of Things (UPIOT) is a large-scale and wide-ranging intelligent network formed by connecting all kinds of electrical devices and their personnel in the power system through various information sensing devices (such as RFID devices, GPS, laser scanning, and other information transmission sensing devices), and combining with existing network information technology, electronic technology, and artificial intelligence technology. The ubiquitous power Internet of Things involves five levels of terminal information collectors (sensors, RFID, etc.), edge computing, communication networks, cloud platforms, and artificial intelligence [21]. The schematic diagram of the ubiquitous power Internet of Things is shown in Figure 1. The development of the ubiquitous power Internet of Things will further improve the friendly interaction with the user interface and carry out smart power services, energy efficiency management, smart home, and triple play. The ubiquitous power Internet of Things and the smart grid are intertwined and indispensable. As a smart service system that realizes the interconnection of all things in the power system, human-computer interaction, and comprehensive state perception, the ubiquitous power Internet of Things is uniformly promoted by the national power grid and it is expected to accelerate the business expansion of related enterprises.

In this paper, we have deeply studied the application status of wireless sensor networks in the ubiquitous power Internet of Things, challenges, and prospects for future research. In comparison with the current general selection approaches, the main contributions of the proposed work are as follows:

- Provide a review of the basic concepts of power Internet of things and the overall architecture of ubiquitous power Internet of Things.
- Present a comprehensive review of the application statuses of WSNs in ubiquitous power Internet of Things with the aim to represent the state-of-the-art technology by including the most recent approaches.
- Summarize the challenges of WSNs in ubiquitous power Internet of Things.
- Provide a review of the development trends of WSNs in ubiquitous power Internet of Things.

The remainder of this paper is organized as follows.  Section 2 is a literature survey on the overall architecture of ubiquitous power Internet of Things. In Section 3, it is focused on the research on the application statuses of WSNs in ubiquitous power Internet of Things. A review of the challenges of WSNs in ubiquitous power Internet of Things is provided in Section 4.  Section 5 highlights some of the development trends of WSNs in ubiquitous power Internet of Things.  Section 6 presents the conclusions.

## 2. Overall Architecture of Ubiquitous Power Internet of Things

The online monitoring of important operation parameters in various links of the ubiquitous power Internet of Things can strengthen the prediction, prevention, and control of equipment status from the aspects of safety, reliability, adjustability, and antidisturbance and provide intelligent auxiliary decision-making for reliable monitoring of transmission lines and scientific operation and maintenance of distribution links [22, 23], thus further improving the operation level of power grid, strengthening the two-way interaction with users, and expanding new value-added services [24]. The realization of the above objectives relies on thorough the information perception, reliable data transmission, efficient network construction, and intelligent management and data processing analysis technology of massive sensing information [25].

**Figure 2:**
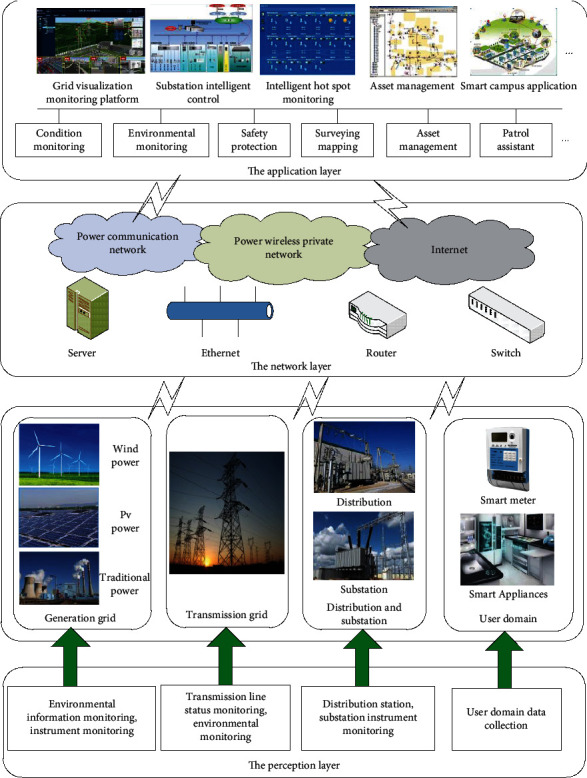
The layered architecture of ubiquitous power Internet of Things.

Wireless sensor network mainly deploys a large number of sensor nodes in the target area, collaboratively perceives and collects various environments or monitoring object information, deeply integrates multiparameter parameters and collaborative processing, and abstracts the state of the environment or object objects [26]. With its unique advantages, wireless sensor network technology can meet the real-time, accuracy, and comprehensive requirements of information acquisition in important aspects such as intelligent power generation, transmission, power transformation, power distribution, and power consumption in various occasions [27]. It can help to achieve effective grid situational awareness and provide effective support for improving the standardized management capabilities of the grid. The ubiquitous power Internet of Things is mainly described in terms of power transmission, substation, power distribution, and power consumption [28]. Through the various kinds of monitoring equipment in the power system, the information acquisition of transmission link's line monitoring, video monitoring, equipment patrol inspection and video monitoring in transformer link, distribution automation in distribution link, equipment monitoring and remote meter reading in power link, and customer relationship are realized. Then, through the power communication system, the information data of each link are transmitted to the management platform to realize information integration, analysis, and processing [29]. For the smart grid, the general architecture of the ubiquitous power Internet of Things can be divided into the perception layer, the network layer, and the application layer, as shown in Figure 2.

For the smart grid, the general architecture of the ubiquitous power Internet of Things can be divided into the perception layer, the network layer, and the application layer [30].

### 2.1. The Perception Layer

The sensing layer is mainly composed of several sensing nodes deployed in each sensing object. The sensing network is built up in a self-organizing manner to realize intelligent collaborative sensing, intelligent identification, information collection and processing, and automatic control of the grid object and the operating environment. Through a variety of new microelectromechanical system sensors, intelligent sensors based on embedded systems, intelligent acquisition equipment, and other technical means, the identification and acquisition of the mechanical state, energy consumption, and environmental state of key equipment in each link of smart grid, such as the power generation, transmission, substation, distribution, power consumption, and dispatch, are completed.

### 2.2. The Network Layer

The perception layer integrates and expands different types of communication networks, such as power wireless broadband, wireless public communication network, wireless sensor network, and power optical transmission network. It realizes the functions of information transmission, routing, and control between the perception layer and the application layer and provides high reliability, high security, and large-scale data transmission services for the perception information. In the application of smart grid, the transmission, convergence, and control of information in power Internet of things are mainly realized by the power communication network, supplemented by the public telecommunication network. The network layer generally includes an access network and a core network. The core network is mainly a power backbone optical fiber network, and the access network is mainly a power fiber access network, a power line carrier, and a wireless digital communication system. The power communication network provides a high-speed bandwidth two-way communication network platform for the application of power Internet of Things.

### 2.3. The Application Layer

The application layer analyses and processes the information and data of the perception layer according to different business needs. The application layer includes application infrastructure, middleware, and various applications. The various applications of the application layer involve the production and management of the whole life cycle of the smart grid. Through the use of intelligent computing, pattern recognition, and other technologies, the comprehensive analysis and processing of power grid information and the continuous improvement of the level of intelligent decision-making, control, and service of power grid can be realized, so as to promote more convenient, green, and efficient power.

## 3. Application Statuses of WSNs in Ubiquitous Power Internet of Things

### 3.1. Monitoring of Power Equipment and Lines

Power equipment and line condition monitoring mainly utilizes the increasingly perfect information data platform and various business systems to obtain a large number of equipment, line status, power grid operation and environmental meteorology, and other related data of power equipment operation [31]. Then, based on the association analysis of various data strategies, intelligent learning, valuable knowledge of power equipment, line condition assessment, diagnosis, and prediction from the massive data analysis rules, a state assessment model is established. Thus, the individualized state evaluation, intelligent diagnosis of abnormal state, and fault state of power equipment and lines can be realized, and the health status of power equipment and lines can be grasped comprehensively, timely, and accurately [32]. It provides auxiliary decision-making basis for intelligent operation and inspection of equipment and lines and optimal operation of power grid [33].

#### 3.1.1. Multidimensional Perception Online Monitoring System for Transmission Lines

**Figure 3:**
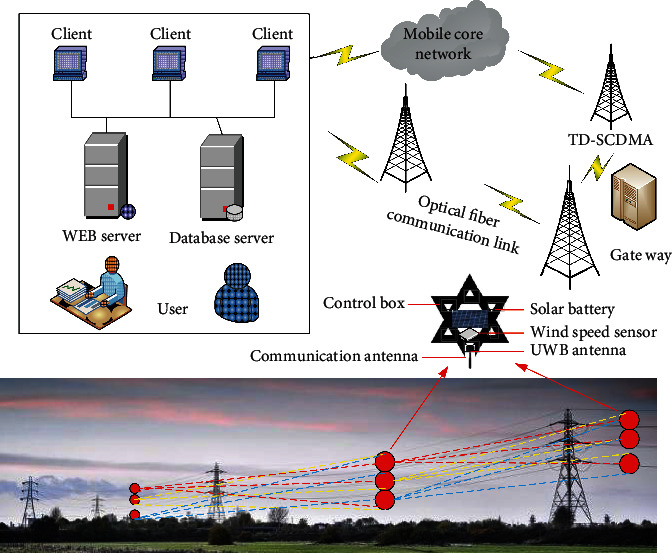
The architecture of the online monitoring system of transmission line based on WSNs.

The high-voltage overhead transmission line is an important part of the power grid. The breeze vibration caused by the breeze and the wind deflection of the wire are the main reasons for the fatigue and breakage of the high-voltage overhead transmission line [34]. The line galloping caused by strong wind conditions will cause great damage to high-voltage transmission lines. At the same time, the line caused by cold weather is covered with ice, and the tower wire is more likely to freeze and the symmetrical pull line icing is often unbalanced, which will lead to the tilting of the tower or even the inversion of towers, which are huge hidden dangers for the safety of the transmission line [35]. By deploying the multifunctional backbone nodes, the MEMS acceleration (gyroscope) sensor node on the entire transmission line, and arranging a leakage current sensor node and a communication backbone node on the high-voltage tower to form a sensor cluster, a plurality of such clusters constitute a linear network [36]. And through the communication, backbone nodes constitute the entire smart grid transmission line online monitoring system. Thereby, the multidirectional visual real-time monitoring and fault warning for various states of the transmission line, such as ice coating, pollution, temperature, galloping, and micrometeorology, are realized. The architecture of the online monitoring system of the smart grid transmission line based on the sensor network is shown in Figure 3.

#### 3.1.2. Transmission, Substation, and Power Distribution Inspection System

There are many types of equipment for transmission, transformation, and distribution in the power grid, and the operation and maintenance workload is large [37]. Usually, a large amount of manpower is required to carry out daily inspections. Through the combination of wireless sensors and RFID tags, the parameters such as operating parameters, abnormal device status, equipment damage, and performance degradation during the daily operation of various power devices are observed and recorded [38]. Through the analysis of the collected data, the hidden dangers are evaluated and warned to avoid the failure of power grid equipment. Based on sensor network technology and RFID radio frequency identification technology, the system realizes the supervision function of the patrol personnel arriving at the scene and patrolling according to the scheduled route. At the same time, environmental information and condition monitoring sensors are added to the system, which can accurately detect the working environment and state of the equipment [39]. It can accurately identify the inspectors and collect the information of the operating environment and working status of the power equipment, thus greatly improving the quality of the inspection work.

#### 3.1.3. Electrical Equipment Status Online Monitoring System

Carry out the state maintenance of power transmission and transformation equipment to improve the utilization rate of equipment, extend the lifetime of equipment, reduce the number of blackouts/blackouts time, and improve the efficiency of transmission. The status monitoring of power transmission and transformation equipment also serves as a diagnostic tool for auxiliary equipment status and plays a huge role in the state maintenance of power transmission and transformation equipment [40]. Through the multisensor integration, multi-information acquisition, information fusion, and antistrong electromagnetic interference technology, the smart grid substation condition monitoring system is established, realizing the real-time monitoring of various equipment and safety protection in the substation. The online monitoring system transmits data to the back-end expert system for analysis and decision-making through the collection of various state quantities of the substation, which can accurately reflect the various states of the substation and provide safety evaluation.

#### 3.1.4. Power Facility Protection and Safety Support Platform

**Figure 4:**
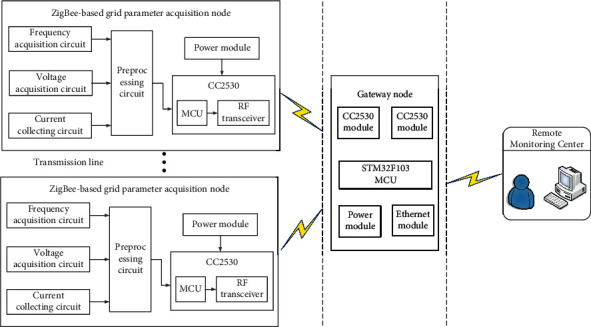
The block diagram of grid parameter acquisition based on WSNs.

Ubiquitous power Internet of Things combined with a variety of sensors to form a collaborative sensing network, wireless sensor network technology. In accordance with certain strategy deployment, vibration, displacement, and voltage change, infrared sensors are installed on outdoor lines, poles, distribution transformers, and other equipment [41]. Collecting and processing on-site information, alarming and cooperative processing can effectively realize the monitoring, early warning, and protection of power grid status information and equipment operation status information. Through a variety of sensors to form a synergistic perception, the effective positioning, monitoring, and early warning of the destruction and theft of the main infrastructure of the power grid (such as equipment, distribution lines, and towers) can be realized, and the power distribution equipment within the monitoring scope can be fully protected [42]. The block diagram of grid parameter acquisition based on wireless sensor network is shown in Figure 4.

Some experts and scholars have carried out a lot of research on power equipment and line condition monitoring. Morales-Velazquez et al. proposed a field-programmable gate array (FPGA) technology for smart meters based on remote monitoring of grid operation. This technology allows the reconfigurable architecture, allows users to select appropriate processing modules according to their applications, so as to realize real-time understanding of system performance and detection of potential faults [43]. In order to overcome the limitation of traditional wired network monitoring technology, a real-time condition monitoring system based on wireless sensor networks (WSNs) is proposed in [44], and the specific application environment is analyzed comprehensively, which provides a strong service guarantee for network monitoring and communication. In the process of line state monitoring of the distribution network, Albu et al. proposed a simplified signal analysis framework for power quality monitoring (PQ) information evaluation methods, which can be effectively used to obtain information of power supply quality of public power grid and has little impact on cost [45]. In the literature [46], the author proposed a concept for continuous waveform recording of power system (EPS) signals by dividing the signals into frames and comparing them. This method is helpful to monitor the power equipment and lines of smart grid in a new environment. In [47], the data cleaning of power equipment monitoring data is cumbersome and easily leads to information loss. The author proposed a data cleaning method based on superimposed denoising and self-coding network. This method has strong ability of denoising and recovering damaged data, can effectively identify and repair outliers and missing information, and can carry out rapid anomaly monitoring in abnormal operation of equipment. In [48], the authors have studied the basis for the reliability of sensor signal acquisition and the accuracy of data processing in the research status of cable equipment, external insulation, generators, power capacitors, and other power equipment status monitoring and diagnosis, and proposed the development direction of equipment monitoring of smart grid in the future. It is reported in [49], the authors aimed at mitigating the fault detection of smart grid and allow the system to self-repair without operator intervention, and an innovative analysis formula for evaluating the reliability of the smart grid distribution system using the Markov method is proposed, which synthesizes the impact of intelligent monitoring on the overall reliability of the system.

**Figure 5:**
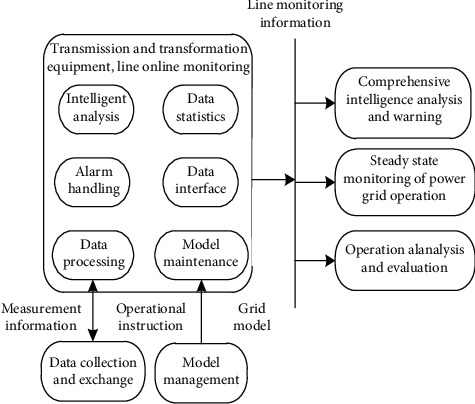
The structural diagram of online monitoring of power equipment and lines.

Based on the main ideas mentioned above, the main structure diagrams of smart grid power equipment and line online monitoring are summarized. The structure diagram of smart grid power equipment and line online monitoring is shown in Figure 5. Online monitoring of power equipment and lines in smart grid mainly includes the following: (1) data acquisition, (2) data processing and storage, (3) integrated alarm processing, and (4) data statistics, model maintenance, intelligent analysis, data interface, and other modules. The system acquires the power grid model from the basic platform of the dispatching system and provides alarm data for the application of power grid such as steady state monitoring, integrated intelligent alarm, operational analysis, and evaluation.

### 3.2. Wireless Automatic Meter Reading of Smart Grid

The traditional electricity consumption statistics use the manual meter reading method, which is usually counted once a month, so that the electricity statistics will have errors. The most important thing is that the power consumption cannot be monitored in real time, which does not meet the requirements of real-time electricity price. The application of the wireless automatic meter reading system based on the wireless sensor network, instead of manual meter reading, can greatly reduce the cost and can monitor the electricity consumption in real-time [50]. The real-time electricity data can be used as the basis for real-time electricity price. Combined with the user's power platform, the user can be well guided to save electricity, reduce the cost of the power grid, and also provide users with preferential treatment to achieve energy-saving and emission reduction. Real-time and accurate power metering can also optimize load forecasting. Combined with the existing power communication network, wireless sensors can accurately monitor the distribution of power usage across the entire grid, providing more accurate and detailed data to the dispatch department.

**Figure 6:**
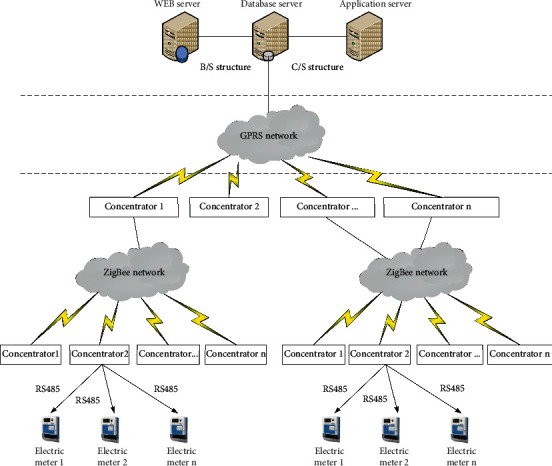
The structure diagram of the meter reading system based on WSNs.

According to the characteristics of the smart home metering system with short communication distance and large number, the wireless sensor network is introduced into the metering system, and the wireless sensor nodes are installed in the home appliances, coordinating each other between household appliances and smart meters in wireless networks. Neto et al. presented several proposed and existing smart utility meter systems as well as their communication networks to identify the challenges of creating scalable smart water meter networks [50]. In [51], an automatic reading system of the traditional household meter is designed on the basis of image processing and advanced DSP system. And to identify the meter reading accurately, a regional average method is proposed to implement the image-scaling in order to avoid the distortion. Mudumbe and Abu-Mahfouz introduced the water management system based on wireless sensor networks, and the system used the IEEE 802.15.4 standard as an open-source application to create a robust and intelligent system [52]. It is reported in [53] that the authors proposed a novel solution for effective smart grid data communication. The authors' proposed scheme achieves the task of collecting data from smart meters by utilizing vehicular ad hoc networks. Most electric power companies employ power meter reader to collect data on the customer's energy consumption, and the author proposed an automatic meter reading system using unmanned aerial vehicles [54]. In [55], the author proposed a solution for water factories in Vietnam using automatic meter reading technology, and the experiments show that this solution can operate well in the real conditions. According to the real-time electricity price information and the user's own needs, it is decided to turn each node on or off. At the same time, the wireless sensor network can be connected with the Internet or GPRS, GSM, etc., and combined into a smart home remote monitoring system. The structure diagram of the smart grid meter reading system based on the wireless sensor network is shown in Figure 6.

From the system architecture, the network is divided into four parts, namely, smart meters, collectors, concentrators, and host computers. As the lowest-level device of the system, smart meters are meters that measure the use of electrical energy. In the system, the collector acts as the terminal node of the network and is responsible for collecting the data information of the electric energy meter and the data information of the electric energy meter of the station area. The concentrator in this system mainly implements two functions. The first is to implement the function of the network coordinator, responsible for setting up the network. The second is to achieve communication with the host computer. The upper computer actually connects the remote automatic meter reading system with people. The upper computer can view the energy data in real time and can also perform data analysis.

### 3.3. Relay Protection of Distribution Network

In the power grid system, the relay protection is an important measure to protect the stable operation of the power system, which is involved in the measurement and control system of the power system. In order to ensure the sensitivity and reliability of the relay protection device, it is required to install differential protection in the small- and medium-sized motor. Due to the high magnetic field strength inside the motor, if the signal transmission line of the sensor is not well shielded or the wiring is not suitable, the motor differential protection may malfunction due to possible electromagnetic noise. The wireless current sensor combined with the coil can be conveniently installed, and there is no conventional current transformer secondary current saturation problem, and wireless communication is used to avoid complicated wiring problems. The sensor sends digital information containing the current amplitude and phase of some of the motors, and these transmitters have time-division multiplexing without interfering with each other. When the frequency of the receiver is set to the same as that of the transmitter, the signal is received and demodulated to obtain the current measured by the sensor on the spot. This information is used to calculate the differential root mean square (RMS) to trigger an alarm or signal.

When the current differential protection is outside the fault, the current transformer error caused by the current transformer saturation is an important factor causing the current differential protection to malfunction. At present, many methods have been proposed for this problem, but these methods are all analyzed and calculated under the condition that saturation has already occurred, and the problem of saturation is not fundamentally solved. Various differential protection systems for coil-based electronic wireless current sensors make it possible to completely solve this problem. Furthermore, the wireless sensor node is coupled with the electronic current sensor, which can replace the wired transmission medium to solve the problem of current signal transmission between the high and low voltage of the electronic transformer. Zhan et al. presented an optimal distributed generation placement method to maximize the penetration level of distributed generation in distribution networks without changing the original relay protection schemes [56]. In [57], the author proposed a detection method for hidden failures based on the features of relay protection systems, and the results indicate that this method can detect hidden failures in relay protection systems and send alarm information. In [58], the author suggested a modified flux-coupling-type superconducting fault current limiter to improve the distributed generation's fault ride-through capability and investigated the relay protection coordination in the microgrid. It is reported in [59] that the author presented an adaptive active relay protection system for the failure state and abnormal operation states, such as short-circuits and overloads, in single-phase Off-Grid systems.

The wireless sensor network provides great convenience for the work of relay protection to collect information and ensures the reliability and stability of this information, and fully exerts the function of relay protection. For the substation protection equipment in which the sensing technology is applied, the parameters are set correspondingly not only in the original transformer current and voltage loop, but also in the position of the transformer itself and the primary and secondary equipment. These include current, voltage, and temperature sensors. These settings will give full play to the monitoring function of the sensor and improve the effect of relay protection.

### 3.4. Life-Cycle Management of Electric Power Assets

In recent years, electric power enterprises have put forward specific management requirements for their asset life-cycle management and actively carry out the linkage correspondence between the stock assets data [60]. The linkage result data are integrated into the integrated platform of life-cycle management of assets, and the indicators are assessed. However, in practical work, there are still problems that cannot be effectively consolidated by the results of previous asset linkage [61].

The application of different levels of RFID technology is carried out throughout the life cycle of the grid assets to realize storage, batch reading, and data transmission of equipment information, realize the precise management of equipment life cycle, and bring good economic benefits. The use of RFID technology can effectively solve the input and output of important assets and business process control tracking in the whole life-cycle management process of the grid, thereby realizing the optimal management of power assets, thereby reducing the error rate of manual operations. This is extremely important for improving the informationization level of asset management. RFID technology can effectively embed the daily management of assets into the asset management system, realize effective management of daily operations of assets, and strengthen the daily supervision of the system, so as to reduce the pressure of daily management of assets. In [62], the author analyzed the fault trends more accurately, and a failure rate model appropriate for general electric power equipment was established based on a nonparametric regression method, improved from the stratified proportional hazards model. Koksal and Ozdemir presented an improved power transformer maintenance plan for reliability centred asset management of the Turkish national power transmission system [63]. In [64], the author proposed a system model that aggregated gridable vehicles and second life batteries together in an intelligent way to provide such backup energy can reduce significant storage costs.

We use the radio frequency identification and identification coding system to automatically identify and locate power equipment, provide technical support for realizing life-cycle management of power assets, improve operational efficiency, and improve management level. The intelligent wireless sensor is installed on the transformer to calculate the cumulative running time and the actual load level; the smart wireless sensor installed on the circuit breaker can automatically record the number of circuit breaker actions, overcurrent conditions, and contact temperature. The data recorded by intelligent sensors are transmitted to the control center through wireless ad hoc networks, and then the remaining service life of the equipment and the current operation status of the equipment are obtained through cloud computing processing data and intelligent analysis and calculation of expert systems. Faults are detected as early as possible, condition maintenance is done well, service life of the equipment is improved, and the life-cycle management of the power assets of the whole network is achieved.

### 3.5. Fault Location of Power Grid

**Figure 7:**
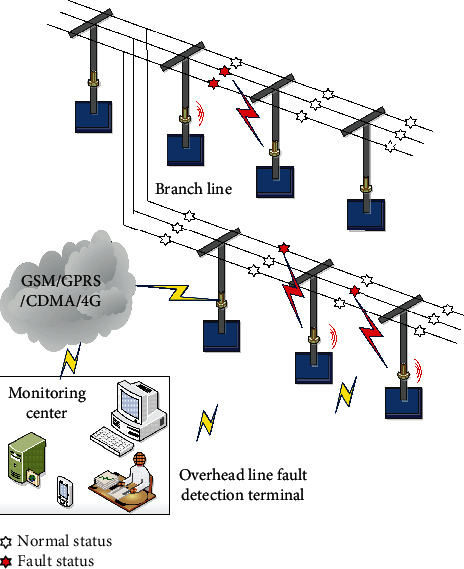
The block diagram of the fault location system for WSNs in smart grid.

In order to ensure the safe and reliable operation of the power transmission network, it is ensured that the background monitoring system can monitor the running status of the transmission line in real time and report the fault location information in time when problems are found on the equipment site. Some experts and scholars use wireless sensor network technology to solve the problem of grid fault location. The block diagram of the wireless sensor network fault location system in the smart grid is shown in Figure 7. WSNs collect data from power grid terminals through nodes and transmit data to user terminals through gateways for data analysis. The reliability and real-time of fault location are guaranteed while reducing manpower, financial, and material resources.

Due to the uneven distribution of power plants and power loads, power transmission through long-distance transmission lines is required, which increases the risk and probability of grid failure. Therefore, after the grid failure, the dispatch center needs to collect accurate fault location information immediately. At present, a large number of universities and state grid enterprises have conducted research on this issue and achieved corresponding results.

Liang et al. proposed a general method for fault location in complex power grid, and the Manhattan distance is adopted to find out the accurate measuring combinations, by which the preliminary fault location is calculated [65]. Aiming at developing a wide-area fault location scheme capable of detecting and identifying erroneous measurements, the proposed method considers the distributed parameter model of the transmission lines [66]. In [67], the author presented two fault location algorithms taking into account the dynamic behavior of wind farm generation. In the first conventional algorithm, fault location is estimated using the recorded voltage and current signals at one end of the line. While the second algorithm is two-end fault location based on the synchronized voltage and current signals from the line ends. To estimate the location of fault in the low-voltage dc microgrid system, a noniterative fault location technique using PPU is proposed in [68]. In [69], the author presented a traveling-wave-based fault location algorithm for hybrid multiterminal transmission systems that consist of one onshore overhead lines and multiple offshore submarine cables. However, the above fault location methods have some shortcomings, such as difficulties in implementation, high economic costs, and weak regional scalability. The wireless sensor network has strong expansibility, convenient real-time and fast. In [70], the author discussed the application prospects of ZigBee in smart grid fault location and the UHV transmission line monitoring. It is reported in [71] that the authors propose a network that allows priority communication: high priority for anomalous events and system control operations and proposes three-layer wireless communication architecture to improve reliability and reduce delays in event notification. In [72], the authors designed an equal-jumping model for the classical Dv-hop localization algorithm of wireless sensor networks in smart grids. The hyperbolic positioning mode is used to calculate the coordinates of unknown nodes to ensure the reliability of the sensor network in fault location.

### 3.6. Fault Diagnosis of Power Grid

**Figure 8:**
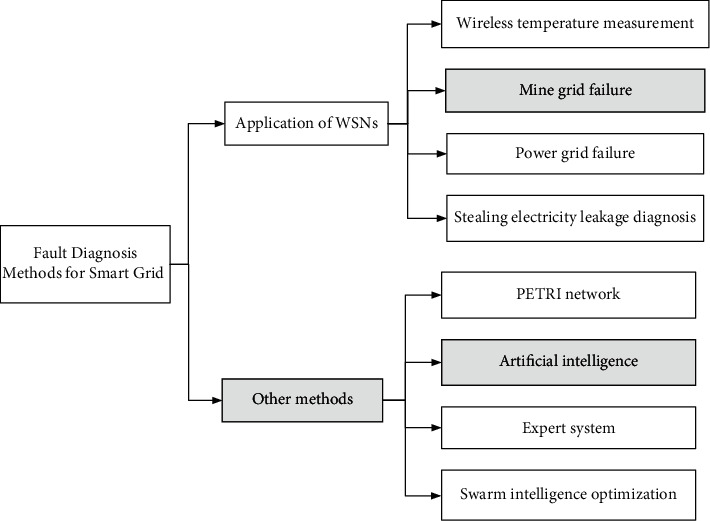
Fault diagnosis method for smart grid.

Wireless sensor networks have been recognized as a technology for seamless, energy-efficient, reliable, and low-cost remote monitoring in smart grid fault diagnosis applications. In these power systems, the required information can be provided to the power facility monitoring center via wireless sensor systems, which can greatly improve inspection efficiency and reduce maintenance costs. Real-time information gathered from these sensors can be analyzed to diagnose problems early and as a basis for taking remedial measures. The intelligent technology of wireless sensor network technology applied to power grid fault diagnosis is reviewed. By combing the research on fault diagnosis methods of multiple intelligent technology fusion, the smart grid fault diagnosis method is shown in Figure 8.

Excessive temperature in electrical facilities is a common cause of facility failure. In [73], the author presented a novel distributed fault detection mechanism for WSNs based on credibility and cooperation, this novel mechanism can achieve high fault detection ratio with a small number of fault diagnoses and low data congestion probability. Aiming at providing a robust system for fault diagnosis, the author used the status of an intelligent electronic device and circuit breakers which can be tripped by any kind of fault. To protect the system from vulnerabilities and different kinds of faults, a multilayered fault estimation classifier, based on the Dominance-based rough set, is proposed in [74]. In [75], the author presented an open-circuit fault detection method for a grid-connected neutral-point clamped (NPC) inverter system. In [76], the author analyzed the currents and voltages sensors fault detection and isolation methods are presented. The proposed method is based on residual generation by models references adaptive systems estimators and virtual flux estimator. In the literature [77], the author proposed a wireless network-based transmission and distribution system monitoring and optimization architecture, which consists of multiple intelligent wireless transformer sensor nodes, intelligent control stations, intelligent transmission line sensor nodes, and intelligent wireless user sensor nodes. The software module also combines different data aggregation algorithms required for different paths of the power distribution system. There are many fault diagnosis methods for smart grid, but for the combination of the above single methods to optimize the diagnosis method, multi-information fusion is bound to be the hot direction of the future development of power grid diagnosis. In the literature [78], Castaño et al. presented the development and implementation of quality monitoring framework based on a model-driven approach using embedded artificial intelligence strategies. A generalized fuzzy clustering C-means method for predicting a quality indicator, represented by surface roughness of manufactured components, is presented for specific manufacturing process. In the literature [79], the author presented a fault prediction approach and load forecasting approach. The approach of data analytics and deep learning for smart grids (SGs) and their applications are proposed in this paper, which seeks to provide a transition to future energy systems while providing significant knowledge of existing smart grids. In [80], the author introduced the concept and status quo of the above three methods, summarizes their potential for application in smart grids, and provides an overview of the research work on their application in smart grids. The principles of the Industry 4.0 are highlighted, by giving emphasis to the features, requirements, and challenges behind Industry 4.0. In addition, a new architecture for AIA is presented in [1].

### 3.7. Summary of Application Status

**Figure 9:**
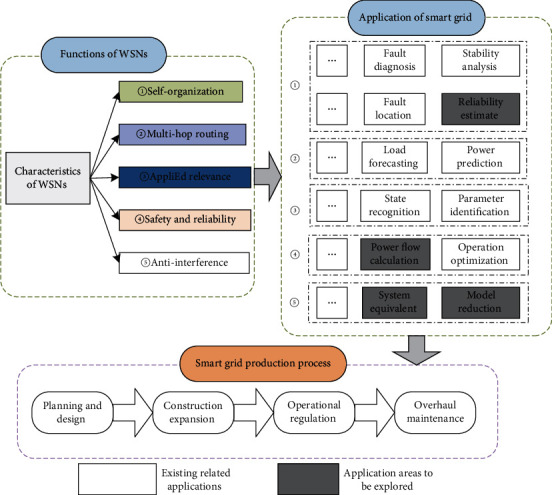
Application of WSNs in ubiquitous power Internet of Things.

It can be seen from the above literature that wireless sensor networks have broad application prospects in smart grids and use wireless sensor network technology to realize the intelligentization of power grids in all aspects of power generation, transmission, and power consumption. In the power generation, wireless sensor network technology is used to realize online monitoring of the operating unit, improve the safety and stability of the unit, and realize the state detection and condition maintenance of the power generation equipment. In the transmission and transformation, wireless sensor network technology can effectively monitor the transmission line and substation equipment online, ensure the safe operation of transmission lines and substation equipment, improve transmission efficiency and transmission reliability, and achieve automatic diagnosis. In the power distribution link of the power grid, wireless sensor network technology is used to realize online monitoring and early warning of the status of the distribution network, so as to realize the rapid positioning of distribution line faults and ensure the safe and stable operation of the distribution network. In the power consumption of the power grid, wireless meter reading technology based on wireless sensor network technology can be used to monitor power usage in real time, optimize load forecasting and grid load scheduling, and guide users to save electricity and prevent electricity from being stolen [81]. The characteristics of the wireless sensor network and its application in various production links of the power system are shown in Figure 8. The application research of the smart grid is gradually deepened, and the matching between the wireless sensor network and the smart grid application scenario needs to be further explored. For example, in the aspect of reliability evaluation, the use of historical operation data of wireless sensor network monitoring components, equipment, and systems can effectively guide the functions of power equipment aging monitoring, life prediction, and fault identification and can also improve the accuracy of power system reliability evaluation [82]. The application of wireless sensor networks in ubiquitous power Internet of Things is shown in Figure 9. The network performance comparison of WSNs in UPIOT is shown in Table 1. In Table 1, the performance parameters of ubiquitous power Internet of Things is provided, mainly including network efficiency, transmission latency, complexity, load balancing, energy utilization, network lifetime, algorithm complexity, and other factors. The system reliability analysis of ubiquitous power Internet of Things is shown in Table 2. The system reliability parameters of ubiquitous power Internet of Things, mainly including network connectivity, availability, invulnerability, scalability, throughput, packet loss rate, robustness, reliability, and other factors, are shown in Table 2.

## 4. Challenges of WSNs in Ubiquitous Power Internet of Things

At present, some applications of wireless sensor networks have been explored in the power industry, but there are some problems, such as single business-oriented applications and lack of top-level planning and design for unified applications of ubiquitous power Internet of Things. Moreover, the application environment of the power wireless sensor network is complicated, and the communication technologies involved in the application are diverse, the protocol is complicated, and the data collaborative processing is difficult. For the specific application of power grid transmission and distribution system, the application of the wireless sensor network in power grid has some challenges in five aspects: transmission line inspection, distribution site operation supervision and intelligent patrol inspection, intelligent power information acquisition, intelligent power service construction, and user service technology and equipment.

- In the online monitoring of transmission lines, the deployment of wireless sensor networks will face severe communication networking technology challenges as transmission lines extend in a linear manner. In addition, most of the high-voltage transmission lines are installed in inaccessible places, and the lines are long and narrow. It is necessary to realize multihop wireless network protocols under the condition of ensuring low energy consumption, which has great technical challenges. Another technical constraint is the power supply problem of online monitoring devices. In addition to the serious electromagnetic interference caused by the transmission system transmission, the wireless sensor network is in a strong electric field environment, and it is also easy to couple to generate high induced voltage and penetrate the burnt equipment. This also poses a severe challenge to the transmission line monitoring equipment.
- In the field of power distribution site operation supervision and intelligent inspection, although the existing system already has the level of intelligence, the application of RFID tags in the existing system is limited to equipment identification and does not have the functions of confirming object status, matching work procedures, and recording operation process, and cannot achieve the ultimate goal of reducing the risk of misuse and potential safety hazards. In addition, the video sensor of the same type of system is not used, and the real-time interaction between the dispatching command center and the field operators cannot be realized. The inspection work (such as the supervision mechanism for the inspection personnel and the automatic information collection process) still needs to be further improved to achieve the purpose of effective, reliable, and safe inspection.
- In terms of intelligent power consumption information collection, the current power consumption information collection and transmission technology has preliminary application, but there are still problems of reliability, availability, maintainability, and practicality. A single communication technology cannot solve the problem of reliable transmission of power usage information in different areas of electricity.
- In the construction of intelligent electricity service, the communication network resources for the customer side are insufficient, the interaction functions between various systems and users are not sufficient, and the requirements for intelligent power service cannot be fully adapted. The system functions need further development and integration.
- In terms of user service technology and equipment, the original system construction is less standardized, and the technical solutions and functions are greatly different. The terminal equipment is diverse in form, the level of intelligence is not high, and the functions are limited.

## 5. Development Trends of WSNs in Ubiquitous Power Internet of Things

**Figure 10:**
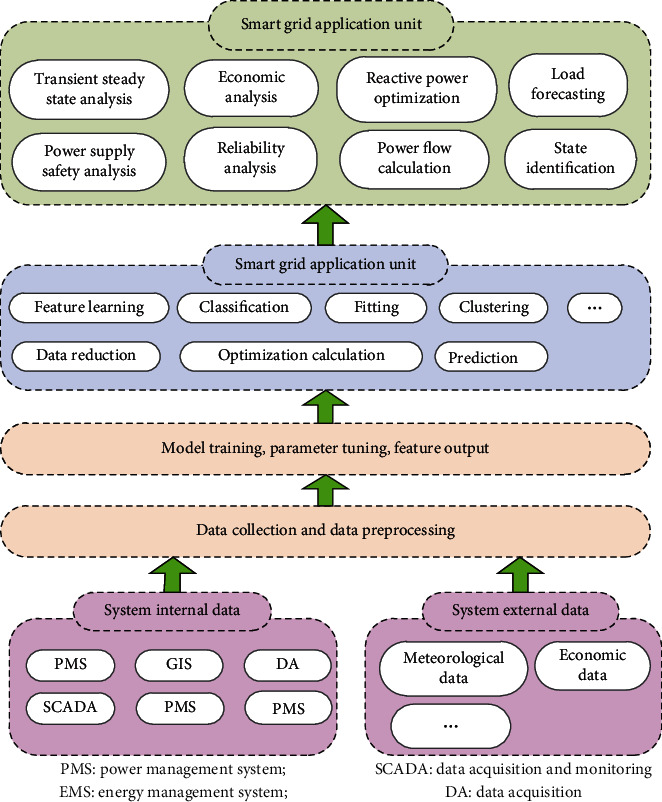
The technical framework of WSNs in ubiquitous power Internet of Things.

Wireless sensor networks have many distinctive features: (1) peer-to-peer network, data-centric; (2) modularization of hardware structure, simple and convenient system construction; (3) dynamic change of network topology; (4) strong transmission capability, low packet loss rate; (5) high energy efficiency; (6) strong anti-interference ability; (7) multihop routing. The application framework of the wireless sensor network in ubiquitous power Internet of Things can be summarized as data acquisition, data preprocessing, model training, parameter tuning, feature output, function, application unit, and so on. The technical framework of WSNs in ubiquitous power Internet of Things is shown in Figure 10.

At present, the ubiquitous power Internet of Things is developing towards high proportion of renewable energy, high proportion of power electronic equipment, multienergy complementary comprehensive energy utilization, and deep integration of physical information. Therefore, it is faced with new energy consumption, safe operation of large power grid, multienergy and high efficiency cascade utilization, information security protection, and intelligent fault diagnosis of equipment. Self-organizing, multihop routing, application correlation, security reliability, and anti-interference based on the technology of wireless sensor network, and its application in relay protection network development, smart substation application research, power grid catastrophe application research, power system security protection and deep-seated ubiquitous Internet of Things are prospected.

### 5.1. Networked Development of Relay Protection

With the rapid development of Internet technology, information technology has been applied to various fields of power grid. In the relay protection technology, it should also be fully integrated with the network technology to achieve information sharing and information transmission by means of the network technology, so that the speed of information transmission is greatly improved, and the effectiveness and accuracy of information can be guaranteed. In addition, the networking of substation can promote the networking of relay protection. The power equipment is connected by wireless communication network technology, the power information can be transmitted efficiently, which provides great convenience for power work, and the relay protection system is more widely used.

### 5.2. Application Research of Intelligent Substation

Intelligent substation is an important foundation and support for a strong smart grid. The intelligent substation is made up of advanced, reliable, energy-saving, environmentally friendly, and integrated equipment. It transmits information by high-speed network communication platform and automatically completes basic functions, such as information collection, measurement, control, protection, measurement, and monitoring, and supports advanced application functions such as real-time automatic control, intelligent adjustment, online analysis and decision-making, and collaborative interaction. Wireless sensor network technology integrates the intelligent sensor technology, the distributed information processing technology, and the wireless communication technology. Different sensors are used according to the practical application detection needs. According to the communication delay and bandwidth requirements in practical application, different radio frequency communication technologies can be adopted. It is very suitable for monitoring nonelectrical parameters in smart substation. The construction of smart substation can be greatly accelerated by using wireless sensor network technology.

### 5.3. Application Research of Power Grid Disaster

The primary goal of the smart grid is to improve its safety and reliability. The main causes of natural disasters causing large-scale power outages in the power grid are failure of the longitudinal protection channel, failure of the main protection, destruction of the power infrastructure, and changes in the protection caused by the grid. At present, most of the existing power communication networks in the world use wired methods for communication. Even a small number of wireless communication networks rely on base stations to transmit information, that is, the existing power communication networks are inseparable from basic communication devices. Under the natural disasters, basic communication devices are very fragile and easily cause network failure. Wireless sensor networks have the characteristics of self-adaptation, self-organization, and multihop. As an effective means to cope with power grid disasters, it is extremely urgent to introduce a new power communication network.

### 5.4. Network Security Protection of Power System

The smart grid is a typical information physical fusion system. It uses a large number of sensing devices and complex communication networks to form a complex system of real-time sensing, information services, and dynamic control. Deep information flow interaction will make the power system face more potential threats. The network attack of the power system has the characteristics of strong concealment and long latency. Although it does not directly damage the primary equipment, it can destroy the secondary system to achieve the purpose of attacking the physical power grid. The wireless sensor network technology can automatically identify the characteristics of the network attack and can be used for malware detection and intrusion detection to realize network security protection of the power information physical system. Compared with traditional machine learning methods, wireless sensor network technology can effectively improve the detection efficiency and correct rate of network security attacks.

### 5.5. Deep-Seated Ubiquitous Power Internet of Things

Ubiquitous power Internet of Things is a kind of intelligent service system with comprehensive state perception, efficient information processing, and convenient and flexible application, which makes full use of modern information technology and advanced communication technology, such as mobile interconnection, artificial intelligence, to realize interconnection, and human-computer interaction in all aspects of the power system. It includes a four-layer structure of the sensing layer, the network layer, the platform layer, and the application layer. Through the extensive application of information technology and intelligent technology such as big data, cloud computing, Internet of Things, mobile interconnection, artificial intelligence, block chain, and edge computing, all aspects of resources are brought together to provide sufficient and effective information and data support for planning, construction, production and operation, management, comprehensive services, new business model development, and enterprise ecological environment construction.

## 6. Conclusion

In this paper, the core technologies of wireless sensor network technology and ubiquitous power Internet of Things are studied in depth, and the power communication network is extended to the production site and users of power grid. It has important guidance and demonstration value for constructing ubiquitous sensing network covering various nodes of grid production, operation, management, and service. On the one hand, the ubiquitous sensing power Internet of Things achieves coverage of more nodes and can comprehensively improve the overall perception, data collection, and service interaction capabilities of grid production, grid services, and grid management. On the other hand, the construction of power Internet of Things and the development of a large number of sensors, terminals, and systems for the collection of the Internet of Things provide a convenient means for data collection, greatly reducing the cost of perceiving various types of data perceived by the power Internet of Things and laying a technical device and the network foundation for the large-scale development and application of large-scale data acquisition and data in the smart grid.

With the continuous development of the scale of the ubiquitous power Internet of Things and the accumulation and increase of the data collection scale, the follow-up work will further develop and study the cross-professional integration, deep sharing, and accurate user service technologies based on the data collected by the power Internet of Things, which can provide more scientific, efficient, and intelligent decision support for the production, operation, and management of the power grid.

## Figures and Tables

**Table 1:** Network performance comparison of WSNs in UPIOT.

Method	Efficiency	Latency	Complexity	Load balancing	Energy utilization	Network lifetime	Algorithm complexity
[9]	Moderate	Small	Low	Moderate	Moderate	High	Low
[18]	High	Small	Moderate	Good	Good	Moderate	Moderate
[19]	High	Moderate	High	Moderate	Good	High	Low
[10]	Moderate	Moderate	Moderate	Good	Moderate	Moderate	Moderate
[21]	Low	Moderate	Low	Moderate	Low	Low	Low
[22]	High	High	Low	Moderate	Moderate	Moderate	Moderate
[23]	High	Moderate	Moderate	Good	Moderate	Moderate	Low
[24]	High	Moderate	Low	Moderate	Good	Low	Moderate
[26]	Low	Small	Moderate	Good	Moderate	Moderate	High
[27]	High	Small	High	Moderate	Moderate	Moderate	Moderate
[28]	Low	Moderate	Moderate	Low	Moderate	Low	High
[29]	High	Moderate	High	Moderate	Good	High	Low
[30]	Low	Small	Moderate	Good	Moderate	Moderate	Low
[38]	High	High	Low	Moderate	Moderate	Low	High
[44]	High	Moderate	Moderate	Moderate	Good	High	Moderate

**Table 2:** System reliability analysis of UPIOT.

Method	Connectivity	Availability	Invulnerability	Scalability	Throughput	Packet loss rate	Robustness	Reliability
[9]	High	High	Moderate	Moderate	Moderate	High	Low	Moderate
[18]	Low	Small	Moderate	Moderate	Moderate	Moderate	Moderate	High
[19]	Moderate	Moderate	High	Moderate	Good	High	Low	High
[10]	High	Small	Moderate	Good	Moderate	Moderate	Moderate	Moderate
[21]	Low	Moderate	Low	Moderate	Low	Low	Low	High
[22]	Moderate	High	Moderate	Moderate	Moderate	Moderate	Moderate	Moderate
[23]	Moderate	Moderate	Moderate	Good	Moderate	Moderate	High	Moderate
[24]	High	Small	Low	Moderate	Good	Low	Moderate	Low
[26]	Low	Moderate	Moderate	Good	Moderate	Moderate	High	Moderate
[27]	High	Moderate	High	Moderate	Good	High	Moderate	High
[28]	Moderate	Small	Moderate	Good	Moderate	Moderate	Moderate	Moderate
[29]	High	Moderate	High	Moderate	Good	High	High	High
[30]	Low	Small	Low	Moderate	Low	Moderate	Moderate	Moderate
[38]	High	High	Moderate	Good	Moderate	High	High	Moderate
[44]	High	Moderate	Moderate	Moderate	Moderate	Moderate	Moderate	High

## Data Availability

The data used to support the findings of this study are included in the article.
